# Imaging Diagnosis and Management of Carpal Trauma and Instability—An Illustrated Guide

**DOI:** 10.3390/life13071426

**Published:** 2023-06-21

**Authors:** Chukwuemeka K. Okoro, Matthew R. Skalski, Dakshesh B. Patel, Eric A. White, George R. Matcuk

**Affiliations:** 1 Department of Imaging, Cedars Sinai Medical Center, Los Angeles, CA 90048, USA; chukwuemeka.okoro@cshs.org; 2 Department of Radiology, Palmer College of Chiropractic—West Campus, San Jose, CA 95134, USA; docskalski@gmail.com; 3 Department of Radiology, Keck School of Medicine, University of Southern California, Los Angeles, CA 90033, USA; dakshesh.patel@med.usc.edu (D.B.P.); eric.white@med.usc.edu (E.A.W.)

**Keywords:** wrist, carpal, instability, trauma, musculoskeletal

## Abstract

Understanding the subtle signs of carpal instability and other unique injury patterns in the wrist is a critical skill for radiologists. Proper patient management and outcomes are directly dependent on the accurate interpretation of wrist imaging studies. This review will provide a detailed overview of typical imaging features of carpal trauma and instability, management, and complications, using multimodality imaging and original medical illustrations. A detailed overview of the osseous, ligamentous, arterial anatomy of the wrist, arcs of Gilula, and zones of vulnerability will be provided. Carpal fractures, dislocations, special radiographic views, and imaging pearls will be discussed. Instability patterns and the myriad of associate abbreviations (CID, CIND, CIC, CIA, VISI, DISI, SLD, LTD, MCI, SLAC, SNAC) will be clarified. Expected outcomes, potential complications, and management will be reviewed.

## 1. Introduction

The carpus is a complicated structure composed of multiple anatomic structures which coordinate together for proper biomechanical function. With so many integral structures allowing for a comprehensive range of motion, direct trauma and injury to any of these structures may lead to wrist instability or dysfunction, which can eventually progress to disability [1].

Investigation of the carpus following trauma and instability symptoms presents a challenge for radiologists, given the complexity of the involved structures and the importance that the report has for patient outcomes. Proper patient management and outcomes are intrinsically tied to the accurate interpretation of wrist imaging studies. Recognition of carpal instability and its associated fractures on initial imaging studies in the acute setting is paramount to the diagnosis and management of these injuries, although diagnostic wrist arthroscopy also plays an important role. Failure to identify these injuries can contribute to long-term complications, including but not limited to degenerative arthritis, nonunion, and even avascular necrosis [2]. These complications are the main contributors to patient reports of continued pain, discomfort, and disability. However, understanding the biomechanics of the carpus is difficult as many articles have conflicting details [3]. This further puts emphasis on the value of fully comprehending the carpal anatomy to better appreciate the complexity of the injury patterns of carpal trauma, which include osseous, ligamentous, and arterial components.

In this article, we discuss the clinically relevant anatomy of the carpus, illustrate common carpal fracture and dislocation patterns on imaging, detail imaging features of carpal instability, and review expected clinical outcomes, potential complications, and common management strategies.

## 2. Carpal Anatomy

**Figure 1:**
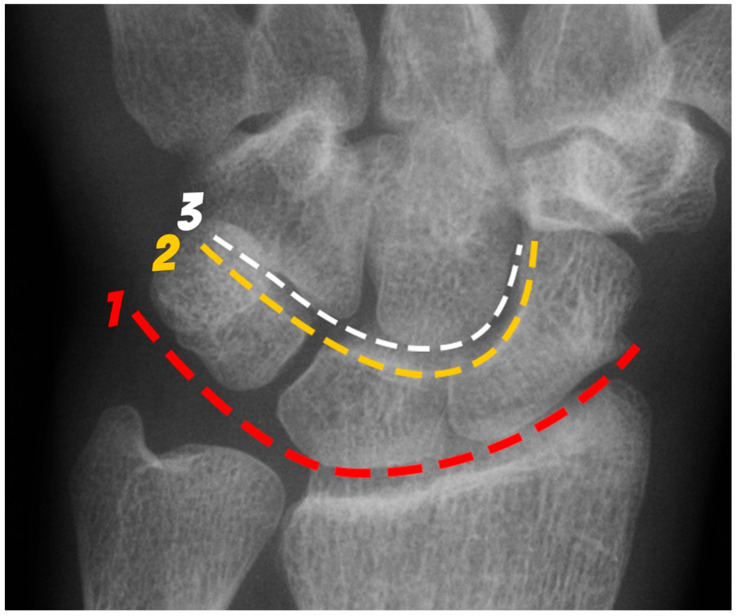
PA radiograph of the wrist depicting the arcs of Gilula. The 1st arc (red) outlines the proximal surfaces and the 2nd arc (yellow) the distal surfaces of the scaphoid, lunate and triquetrum. The 3rd arc (white) outlines the proximal surfaces of the capitate and hamate.

The wrist includes the distal ends of the radius and ulna, eight carpal bones, and proximal bases of the metacarpal bones [4]. The carpal bones are divided into a proximal row and distal row. The proximal row, consisting of the scaphoid, lunate, triquetrum, and pisiform bones, is considered an intercalated segment between the radius and distal carpal row. The proximal row is central to maintaining wrist stability by balancing movement and regulating the forces sent to and from the hand and the forearm. The distal row consists of the hamate, capitate, trapezoid, and trapezium bones, which form a more stable and rigid alignment than the proximal row as they function to support the bases of the metacarpals [5]. The arcs of the Gilula are used on PA wrist radiographs as part of assessing the normal alignment of the carpus (Figure 1). There are three arcs:

- Proximal (first) arc—smooth line along the proximal surfaces of the scaphoid, lunate, and triquetrum.
- Middle (second) arc—smooth line along the distal surface of the scaphoid, lunate, and triquetrum.
- Distal (third) arc—smooth line along the proximal surface of the capitate and hamate, closely paralleling the second arc.

In a biomechanically normal wrist, there should be no irregularities in the contour of these arcs when traced. Any step-offs/disruptions in these lines suggest a ligamentous injury or fracture at the site of the broken arc [6]. Exceptions include two normal variants, one where the triquetrum is shorter than the lunate creating a step-off of the first arc but normal second arc, and a second when a type II lunate can cause a bilobed appearance of the second and third arcs between the capitate and hamate [7].

The intrinsic (interosseous) and extrinsic (capsular) ligaments of the wrist are essential for stability and mobility. Intrinsic ligaments are defined as those attachments that connect one carpal to another. These include the scapholunate ligament, lunotriquetral ligament, midcarpal ligaments, and distal carpal row interosseous ligaments, of which the scapholunate and lunotriquetral are the most important. In function, the scapholunate ligament resists volar flexion of the scaphoid and dorsal extension of the lunate. The lunotriquetral ligament balances proximal carpal row motion in the opposite direction. Extrinsic ligaments are defined as those that connect the carpal bones to adjacent structures, such as the metacarpals and distal radius and ulna. The extrinsic ligaments provide gross stability to the wrist, whereas the intrinsic ligaments contribute more towards fine wrist motion [8].

The carpal bone blood supply to the scaphoid, lunate and capitate is unique in that the arteries enter the distal half of each bone. This is important as this puts the proximal portion of the bone at risk for ischemic injury and avascular necrosis when the bone is fractured. The arterial anatomy consists of the radial and ulnar artery branches in addition to the anterior interosseous branches supplying the arches. The radial artery traverses the lateral aspect of the wrist and provides blood supply largely to the dorsal structures of the hand. The ulnar artery traverses along the medial aspect of the wrist in the Guyon Canal, composed of the deep palmar branch that continues in the Guyon Canal while the superficial palmar branch that exits the canal and crosses the surface of the hypothenar muscles and deep to the palmar aponeurosis, providing blood supply to the digits. Although variable in its presentation, the superficial branch is particularly vulnerable to injury and can be compressed against the hook of the hamate [9].

## 3. Imaging Modalities

A basic standard radiographic series of the wrist includes PA, lateral, and oblique views. These views are sufficient to identify most pathology, however the use of stress radiographs can provide additional valuable information. Commonly obtained stress views of the wrist include clenched fist and ulnar deviation views, both of which allow for a more sensitive dynamic assessment of scapholunate instability. The ulnar deviation view also tensions the scaphoid into a more upright position for better visualization of its long axis, similar to an angulated scaphoid view.

Lateral wrist radiographs provide a wealth of information in the assessment of carpal alignment but are susceptible to suboptimal projection at the time of imaging which frequently limits assessment. A well-positioned lateral wrist radiograph should demonstrate the volar surface of the pisiform midway between the ventral surface of the capitate and distal scaphoid. When this has been accomplished, intercarpal alignment is most accurately demonstrated. In this scenario, with the wrist in the neutral position (neither flexed nor extended) a line that bisects the center of the articular surface of the radius drawn along the long axis of the radius should align with the axis of the lunate and capitate. Deviation of the lunate or capitate away from this line is an indicator of carpal instability [2]. When this concern arises, the scapholunate and capitolunate angles should be measured. The scapholunate angle is determined by a line along the long axis of the scaphoid (roughly parallel to a line tangential to its ventral surface) and a line drawn along the mechanical axis of the lunate which bisects its proximal and distal surfaces (perpendicular to the tips of the distal cup). The normal limits of the scapholunate angle are between 30° and 60°. The capitolunate angle is determined by the same lunate axis line previously described compared to the long axis of the capitate. The normal limits of the capitolunate angle are between 0° and 30° [10]. Any measurement outside the normal range for these two angles is suggestive of carpal instability.

Plain radiographs are the most common initial imaging modality for identifying carpal fractures and instability, and cross-sectional imaging is usually not required. However, in the setting of uncertainty or negative radiographs, obtaining a computed tomography (CT) or magnetic resonance (MR) imaging can add further details to the trauma evaluation of the wrist. In one study, plain radiographs had a sensitivity and specificity of 69.7% and 83.3% for the detection of carpal fractures in the emergency setting, in contrast to CT scans having a sensitivity and specificity of nearly 100% when used in conjunction with a physical exam and clinical assessment [11]. CT should be obtained with relatively thin slices (2 mm) to cover the area from the distal radius and ulna to the proximal metacarpal shafts. Multiplanar reformations (MPR) images with both bone and soft tissue kernels should also be provided. Viewing the anatomy in 3D volume-rendered reconstructions can be of additional help in recognizing carpal instability patterns but is limited in the detection of carpal fractures in comparison to its 2D counterpart [5]. MRI allows direct visualization of ligamentous tears. Even more, MR arthrography can add additional diagnostic value by determining the presence of otherwise difficult-to-recognize tears of important structures, such as the scapholunate and lunotriquetral ligaments [12,13].

## 4. Carpal Fractures

Traumatic carpal fractures and dislocations are common in young, active patients. These types of injuries cause pain, disability, and decreased productivity. Conventional radiography is still the main imaging modality for evaluating and identifying suspected carpal traumatic injury [13]. However, multi-detector computed tomography is increasingly becoming more utilized, particularly in the following situations:

- Indeterminate initial radiographic findings
- Determination of extent of fracture/dislocation for preoperative surgical planning
- High index of suspicion for carpal fracture with negative initial radiographic findings

Multidetector CT can easily show the extent of carpal fractures and dislocations, often showing fractures that are not visible on radiographs [5]. In addition, pathological conditions and anatomical relationships are better recognized through multiplanar (2D) reformations and volumetric (3D) reconstructions. This information can be easily communicated to orthopedic and trauma surgeons and is essential for surgical treatment and planning.

The most common carpal bone to fracture is the scaphoid. Classically, patients present pain in the anatomical snuff box after a hyperextension trauma. This is a common occult fracture on plain radiographs, with up to 65% going undetected and thus misdiagnosed clinically as a sprain [11]. Inadequate identification of this fracture can lead to complications such as malunion, delayed union, nonunion, and even avascular necrosis, each of which can lead to considerable functional disability [5]. If suspected, a 25° angulated scaphoid view may improve initial radiographic detection rates. However, if a scaphoid fracture is suspected, but the radiographic examination is negative, cross-sectional imaging should be obtained for a definitive assessment, or the patient should be immobilized and re-evaluated with radiographs in 7–10 days when fracture resorption and remodeling may be evident. When CT is obtained to assess for a scaphoid fracture, images should be acquired along the long axis of the scaphoid while the hand is in ulnar deviation for the highest in-plane resolution and then reconstructed using a sharp (bone) reconstruction kernel [3,11]. This technique is also essential in the accurate assessment of fracture healing. The most optimal patient position is with the arm above the head, with the wrist in the prone position. MR has the highest sensitivity and specificity for scaphoid fracture but may lead to difficulty distinguishing between contusions/trabecular microfracture or cortical fractures.

**Figure 2:**
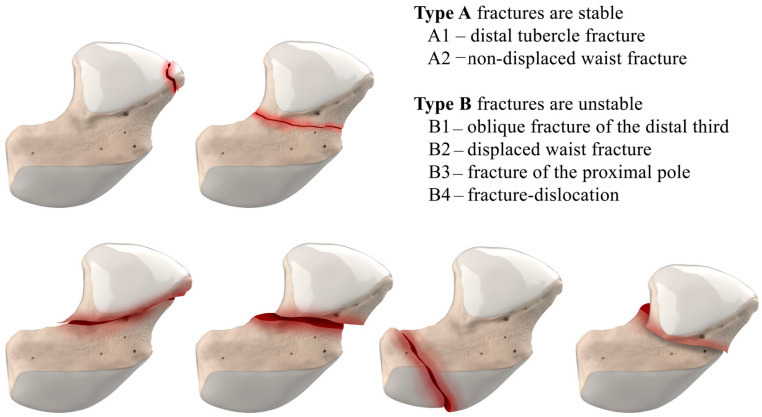
Herbert Classification of scaphoid fractures. Type A fractures are stable, and Type B are unstable.

**Figure 3:**
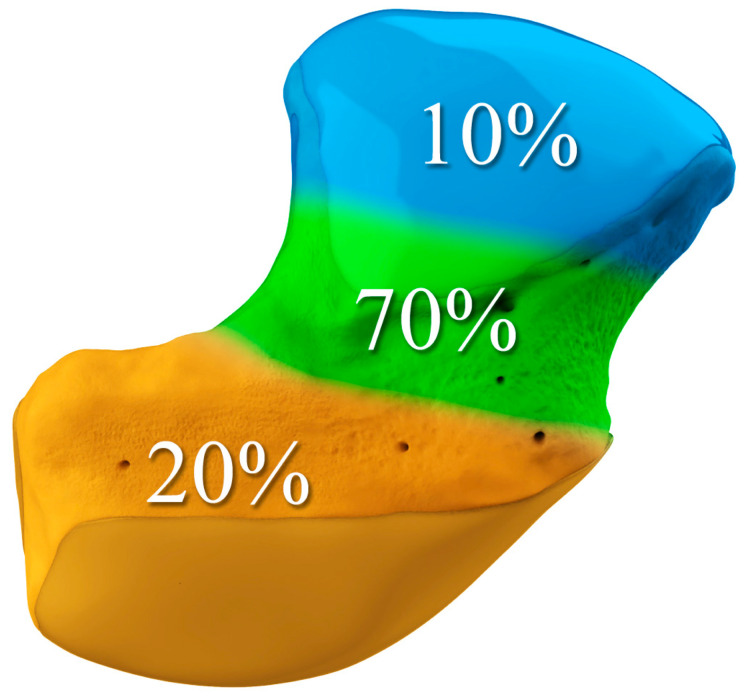
Illustration of the distal, waist, and proximal third sections of the scaphoid, depicting fracture distribution.

**Figure 4:**
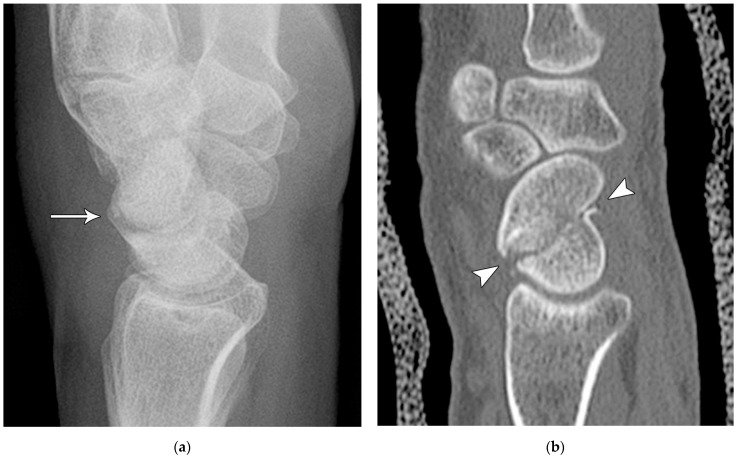
(**a**) Lateral radiograph of the wrist showing Humpback deformity (arrow) after a scaphoid fracture. (**b**) Sagittal CT reformation of the wrist showing the full extent of a humpback deformity with dorsal prominence at the site of the scaphoid fracture (arrowheads); image courtesy of Frank Gaillard, Radiopaedia.org, rID: 18269.

The majority of scaphoid fractures (70%) occur through the waist of the scaphoid, with 10% of scaphoid fractures involving the distal third, and 20% involving the proximal third (Figure 2 and Figure 3) [2,14]. With a scaphoid waist fracture, dorsal prominence at the fracture site due to dorsal angulation is known as humpback deformity (Figure 4). This deformity is important to recognize, as it needs to be more aggressively managed due to high risk of nonunion [15]. Additionally, if the two fragments unite with a humpback deformity, it may further destabilize the wrist. This humpback deformity may also be associated with dorsal intercalated segment instability (DISI), which will be covered in more detail later. 

**Figure 5:**
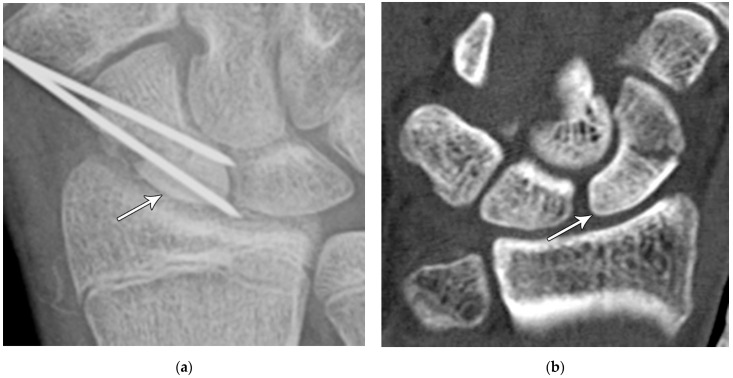
(**a**) PA radiograph showing marked sclerosis (arrow) of the proximal pole of the scaphoid after a scaphoid fracture. (**b**) Coronal reformatted CT in a different patient showing sclerosis (arrow) of the proximal pole of the scaphoid after a scaphoid fracture, indicating osteonecrosis.

**Figure 6:**
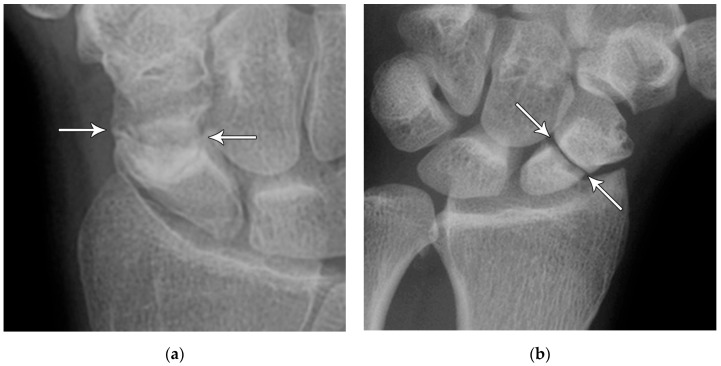
(**a**) PA radiograph showing a transverse lucent line through the scaphoid waist indicating fracture (arrows), with sclerosis and hypertrophic changes of the fracture margins indicating nonunion with pseudoarthrosis. (**b**) PA radiograph of the wrist in a different patient showing a more obvious nonunion, again with sclerosis of the fracture margins (arrows) in an untreated injury.

**Figure 7:**
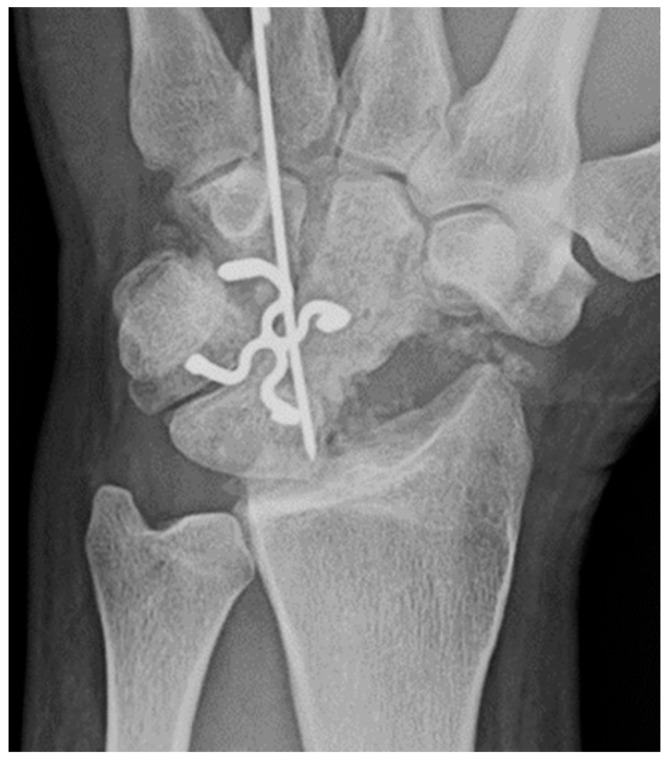
Four corner fusion with scaphoidectomy and surgical staple fusion of the lunate, capitate, hamate, and triquetrum with temporary Kirschner wire fixation.

Common scaphoid fracture complications include avascular necrosis and nonunion [10]. Avascular necrosis is a dreaded outcome resulting from the loss of the proximal arterial supply due to the distal entry point of nutrient vessels. This retrograde perfusion of the proximal pole makes it vulnerable to losing its vascular supply when fractured. Sclerosis of the proximal pole indicates osteonecrosis (Figure 5). With scaphoid nonunion, biomechanical alterations contribute to rapidly progressive degenerative arthritis and instability referred to as scaphoid nonunion advanced collapse (SNAC) [16]. Nonunion occurs in 5–10% of cases and has a higher incidence in displaced fractures (Figure 6). During wrist extension, the distal scaphoid fragment rotates into flexion while the proximal scaphoid fragment extends with the lunate. The resultant abnormal contact between the distal fragment and radius begins the first stage of degenerative collapse. This progresses into the second stage as scaphocapitate osteoarthritis develops and finally to the third stage with progression to capitolunate osteoarthritis and proximal radial migration of the capitate dorsal to the lunate. Treatment of SNAC wrists, depending on the degree and severity of the osteoarthritic collapse, includes scaphoidectomy with four-corner arthrodesis or wrist fusion [17]. Four-corner fusion helps reduce joint pain while maintaining some range of motion, as opposed to total wrist fusion (Figure 7). Another option is proximal row carpectomy with resurfacing capitate pyrocarbon implant placement [18].

**Figure 8:**
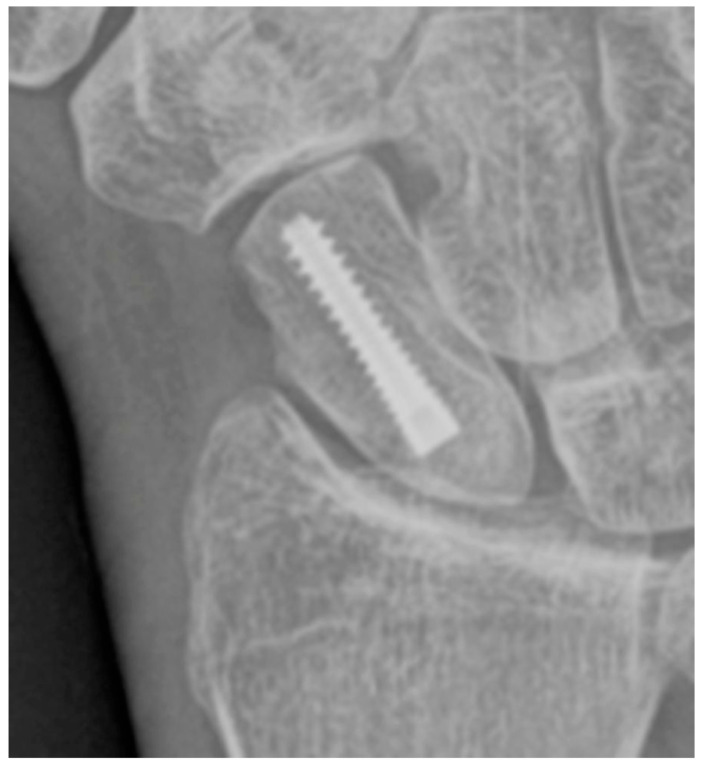
PA radiograph showing surgical fixation of a scaphoid fracture utilizing a variably threaded headless compression screw, allowing it to be countersunk beneath the articular surface, removing the need for retrieval after the fracture has healed. In this case, the screw has been introduced via a dorsal approach, advanced from proximal to distal.

**Figure 9:**
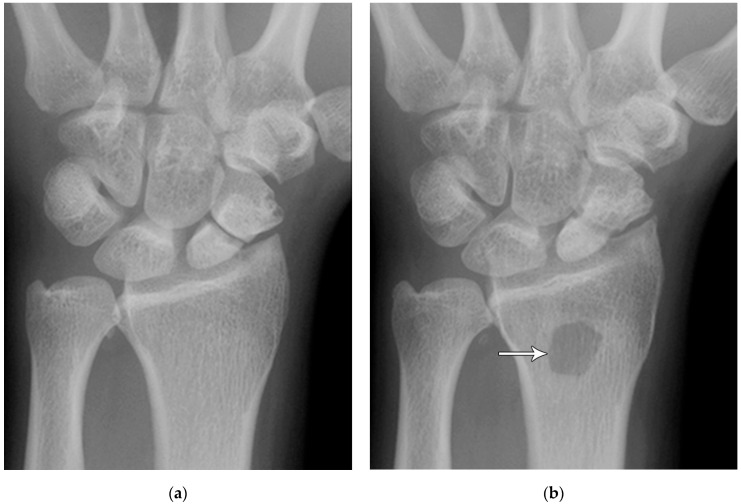
(**a**) Pre-operative wrist radiograph before vascularized bone graft procedure for scaphoid fracture nonunion; (**b**) post-operative wrist radiograph after a vascularized bone graft has been harvested from the distal radius (arrow) and inserted into the fracture nonunion site.

Conservative management of scaphoid fractures consists of cast immobilization across the wrist, including the first metacarpophalangeal joint. This is the most widely used management strategy for scaphoid fractures, particularly when the fracture is stable [2,5,19]. Low rates of morbidity and long-term disability are advantages of a conservative approach; however, this may require a prolonged time to resume work and daily activities of living compared to surgical fixation. The surgical approach involves minimally invasive screw fixation using a compression (e.g., Herbert or Accutrak) screw to allow early mobilization, improving return to activity time over casting. The surgical approach utilizes threaded headless screws (Figure 8), allowing them to be countersunk beneath the articular surface and eliminating the need for removal after fracture healing [17]. Variable threading of the fixation screw promotes compression at the fracture site, facilitating healing. Surgical staple fixation of scaphoid waist fractures is another alternative [20]. Percutaneous screw fixation is contradicted in displaced scaphoid fractures or with nonunion because the fracture may become displaced during the procedure, requiring a subsequent open reduction. A supplementary option for scaphoid fractures with nonunion or sclerosis and/or cystic change is a vascularized bone graft. In these cases, a bleeding surface is re-established at the fracture site for the bone graft containing a vascular pedicle to be incorporated into. The vascular pedicle is typically salvaged from the distal radius (Figure 9a) and inserted into the revitalized fracture gap to re-establish blood flow to the proximal pole (Figure 9b). The vascular pedicle is anastomosed with the radial artery [21].

**Figure 10:**
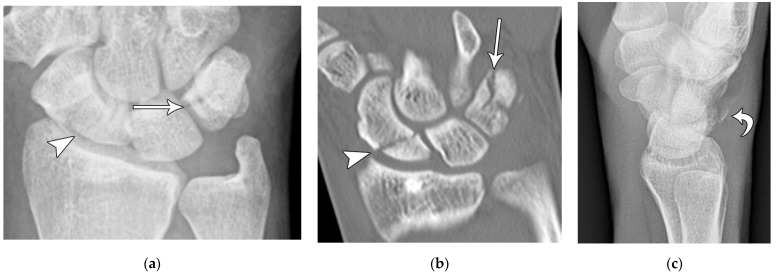
(**a**) PA radiograph of the wrist showing a triquetral body fracture (arrow) and concurrent scaphoid fracture (arrowhead). (**b**) Coronal reformatted CT confirms these findings with greater detail. (**c**) Lateral radiograph of the wrist showing a dorsal cortex fracture of the triquetrum with a tiny, avulsed fragment (curved arrow).

The next most common carpal fracture is the triquetral fracture. Most of these occur on the dorsal aspect of the triquetrum and are best identified on the lateral radiograph at the dorsal aspect of the triquetrum and are best identified as the so-called “pooping duck sign”. These result from compression of the ulnar styloid process and the dorsal aspect of the triquetrum during forceful ulnar deviation and wrist hyperextension [4,5]. Triquetral fractures are subdivided into body, volar cortex, and dorsal cortex fractures, with the latter being the overwhelming majority. Triquetral body fractures (Figure 10a,b) are injuries that are often associated with intrinsic ligamentous injury, such as a lunotriquetral tear. Dorsal cortex fractures (Figure 10c) represent avulsions of the combined attachment of the dorsal radiocarpal and dorsal intercarpal ligaments. Management of dorsal cortex fractures and nondisplaced body fractures consists of 3 to 6 weeks of cast immobilization. If there is concomitant intrinsic ligamentous injury such as a lunotriquetral ligament tear, this injury is unstable and requires surgical stabilization [17].

**Figure 11:**
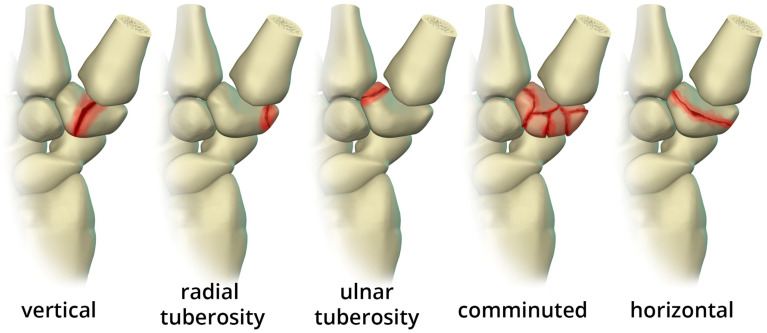
Illustration of the different types of trapezium body fractures.

**Figure 12:**
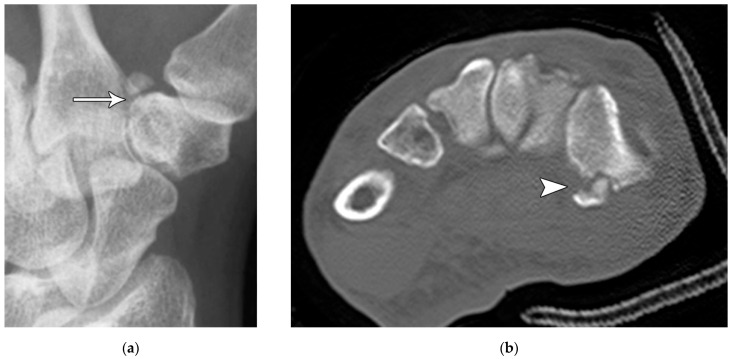
(**a**) Medial oblique hand radiograph showing a well-corticated ossicle in the expected location of the trapezial tubercle, a rare anatomic variant known as os trapezium secundarium. These may represent chronic fracture fragment nonunion. (**b**) Axial reformatted CT in a different patient showing a trapezium ridge fracture (arrowhead).

Trapezium fractures are divided into body and ridge fractures. Body fractures are often associated with fractures of the base of the first metacarpal [11]. These are further subdivided into vertical intra-articular, dorsal, radial tuberosity, ulnar tuberosity, comminuted, and horizontal fractures (Figure 11). Ridge fractures either involve the base or the tip. Ridge base fractures are a result of direct palmar trauma. These can appear similar to the rare trapezium secundarium (Figure 12a), which is an accessory ossicle that is found adjacent to the trapezial tubercle. The trapezium ridge fractures that involve the tip (Figure 12b) are avulsions caused by a sudden tension force applied to the transverse carpal ligament/flexor retinaculum. Management of nondisplaced fractures involves immobilization for 4 to 6 weeks for proper healing. If displaced, trapezial ridge fractures are at risk for nonunion due to pull from the transverse carpal ligament. Surgical fixation or fragment excision are options, particularly in high-performance athletes [17]. Trapezial body fractures that are displaced greater than 2 mm and/or have carpometacarpal subluxation require surgical fixation to prevent long-term osteoarthrosis. 

**Figure 13:**
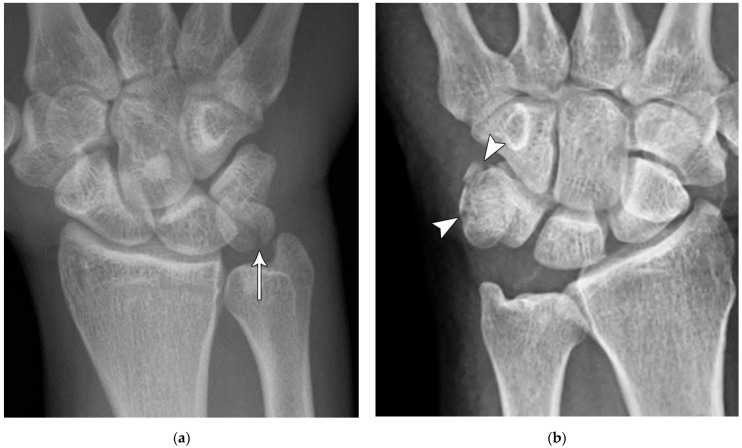
Two examples (PA wrist radiographs) of pisiform fractures with radiographs showing displacement (arrow, (**a**)) and comminution (arrowheads, (**b**)).

The pisiform has the potential to fracture in a multitude of patterns depending on the mechanism of injury. A direct blow is the most common, while a transverse fracture can be seen with resisted hyperextension, as the pisiform exists as a sesamoid within the flexor carpi ulnaris tendon [5,11]. All but the most displaced fracture of the pisiform can be missed on routine radiographs. Additional radiographic views such as the supinated (or semi-supinated) oblique and carpal tunnel view or cross-sectional imaging should be considered in cases with a high index of suspicion (Figure 13). Management includes cast immobilization for 4 to 6 weeks for acute nondisplaced or small avulsion type fractures. Pisiformectomy is reserved for high-performance athletes that want a fast and safe return to sport [17]. 

**Figure 14:**
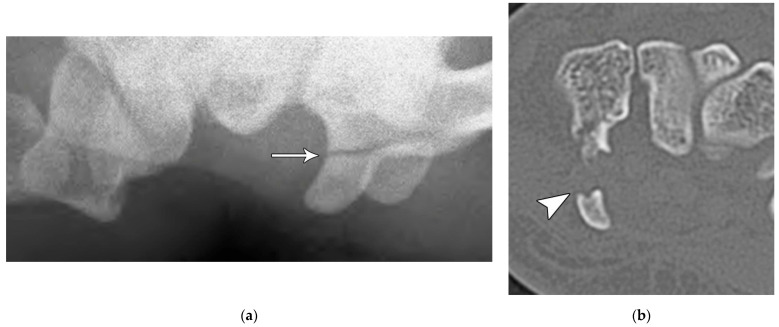
(**a**) Tunnel view hand radiograph showing a fracture through the waist of the hook of the hamate (arrow). (**b**) Axial CT images showing a highly displaced, comminuted fracture of the hook of the hamate (arrowhead).

Like the trapezium, hamate fractures are subdivided into body and hook fractures. Body fractures occur with a variety of fracture patterns without any specific difference in management related to the fracture types [22]. For non-displaced hamate body fractures, treatment with 4 to 6 weeks of cast immobilization is adequate, while displaced hamate body fractures require open reduction and internal fixation to prevent hamatometacarpal subluxation and limited functional recovery. Hook fractures are usually caused by direct impact trauma or from sports where a handle is held, resulting in avulsion fractures at the pisohamate ligament attachment (Figure 14a). Tenuous blood supply to the hook (50%) results in nonunion with waist and tip fractures. Ulnar nerve paresthesia or hand weakness may also possibly occur if the ulnar nerve is injured as it passes around the hook of the hamate (Figure 14b). Management of hook fractures necessitates prompt diagnosis to prevent a variety of complications if treatment is delayed. These include symptomatic nonunion, ulnar neuritis, hypothenar hammer syndrome, and flexor digitorum profundus tendon rupture. With casting for non-displaced fractures, patients should be counseled about the prolonged immobilization required due to poor healing rate, as well as the potential complication of nonunion. For displaced or chronic fractures, as well as those with ulnar nerve compression, excision of the hook is preferred to open reduction/internal fixation because studies have shown that there is no increased restriction in wrist range of motion or grip strength with hook excision [17]. 

**Figure 15:**
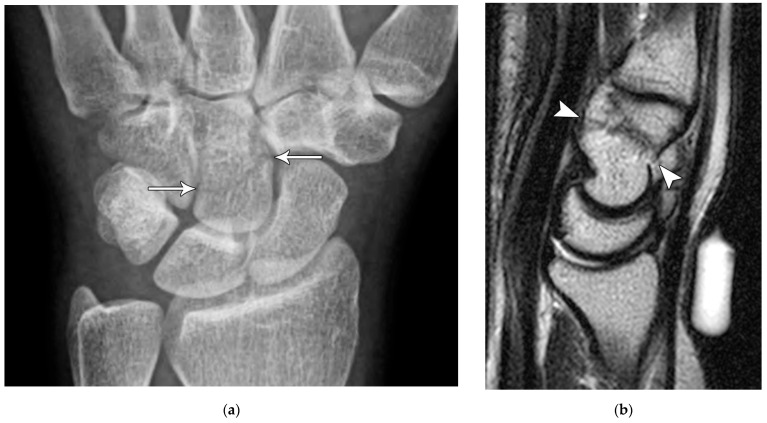
(**a**) PA wrist radiograph showing a transverse capitate body fracture (arrows). (**b**) Sagittal T1 weighted MR image showing a transverse body fracture (arrowheads).

Fractures of the capitate usually occur in conjunction with other fractures. Although isolated fractures do occur, they are quite rare. The proximal pole of the capitate, like the scaphoid, is vascularized via a retrograde blood supply from the distal pole and is consequently vulnerable to avascular necrosis of the proximal component [11]. Capitate fractures are classified as transverse pole, transverse body and neck, vertico-frontal, and parasagittal. Transverse body fractures are the most common (Figure 15). An os styloideum (also known as a carpal boss) represents a common anatomic variant that is occasionally painful and could potentially be misidentified as a fracture fragment. A rare condition involving capitate fracture is Fenton’s syndrome also known as scaphocapitate fracture syndrome [21]. This is characterized by transverse fracture of the scaphoid and capitate, with rotation of the proximal fragment 90° distal to proximal or 180° lateral to medial. A spectrum of concomitant injuries has been reported with this injury pattern, as would be expected. This injury can generally be thought of as a transscaphoid, transcapitate, perilunate fracture-dislocation which spontaneously reduces, causing inversion of the proximal fragment of the capitate. Management of nondisplaced capitate neck fractures is 6 to 8 weeks of immobilization. If pain persists, a CT should be obtained to evaluate for nonunion. Displaced fractures need to be surgically fixated to prevent nonunion and restore normal kinematic anatomy. Given the risk of nonunion in capitate fractures in general, surgical fixation may be indicated to expedite healing and return to play in athletes [17].

**Figure 16:**
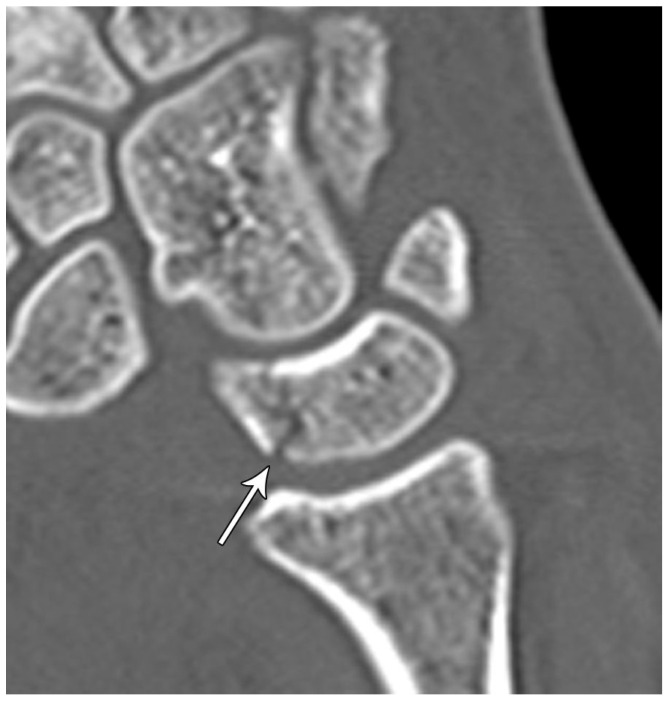
Sagittal reformatted CT of the wrist showing transarticular lunate fracture (arrow).

Lunate fractures are categorized as volar pole, dorsal pole, transverse, osteochondral, and transarticular (Figure 16). Volar pole lunate fractures are the most common and are due to the axial compression from the capitate with the wrist in dorsiflexion and ulnar deviation and additionally may lead to volar intercalated segment instability (VISI) [23]. Like the scaphoid and capitate, the proximal lunate is susceptible to vascular compromise in trauma, with up to 20% of people having only a single palmar nutrient artery supplying the entire lunate. Management of stable, nondisplaced lunate fractures consists of 4–6 weeks of immobilization. Surgical management is indicated for displaced fractures and those with concomitant scapholunate ligament or lunotriquetral ligament injury to preserve the existing anatomy and ensure stability. Chronic fractures with advanced arthritis may even call for partial fusion or proximal row carpectomy [10].

**Figure 17:**
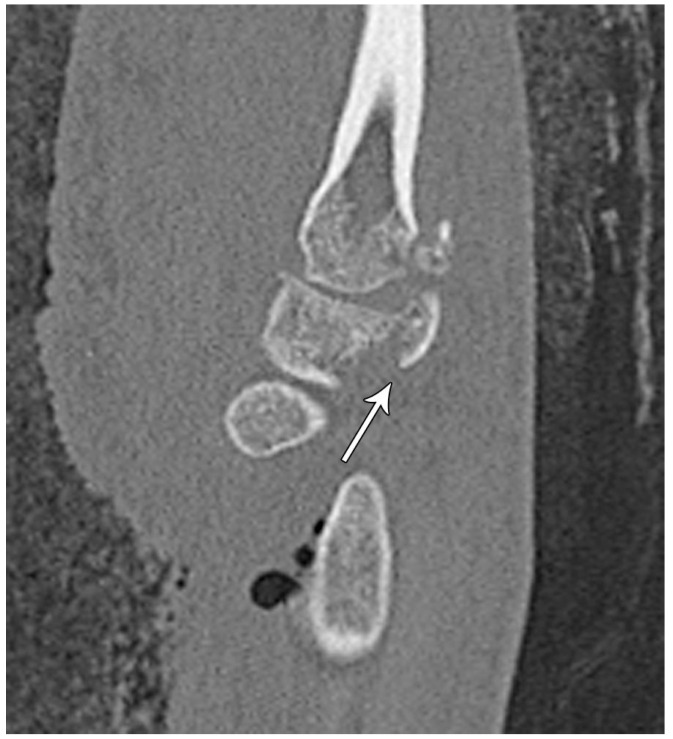
Sagittal reformatted CT of the wrist showing a comminuted fracture of the trapezoid with a dorsally displaced dorsal rim avulsed fragment (arrow) in a patient with a high-energy trauma and multiple other fractures.

The trapezoid is the least commonly fractured carpal bone, as it exists in a relatively protected location [24]. Fracture usually occurs as the result of high-energy trauma, direct trauma, or an axial load through the 2nd metacarpal (Figure 17). Generally, trapezoid fractures occur through the dorsal rim or body. Due to the wedged keystone shape of the trapezoid, combined with the fact that its dorsal ligamentous support is weaker than the volar, trapezoid fractures typically dislocate dorsally. Mildly displaced fractures are treated with casting. Severely displaced and comminuted fractures with articular incongruity require open reduction and internal fixation. Excision of trapezoid fracture fragments is contraindicated, as this leads to subluxation of the second metacarpal and degenerative arthritis [5].

## 5. Carpal Dislocations

Carpal and carpometacarpal dislocations and fracture-dislocations are infrequent but severe injuries, which can be overlooked both clinically and radiographically [8]. This has a significant functional and economic impact, especially since these injuries occur most frequently in working-age young men. A detailed understanding of the normal anatomy and appearance of the carpometacarpal and intercarpal joints is essential to assess for pathology, most notably the scapholunate joint. The normal scapholunate joint space is less than 3 mm. Widening can be subtle, and up to 4 mm may be normal in some patients; however, if a scapholunate injury is suspected, a clenched fist stress view radiograph may be useful for dynamic assessment, as this will make widening of the joint space more conspicuous [5]. 

**Figure 18:**
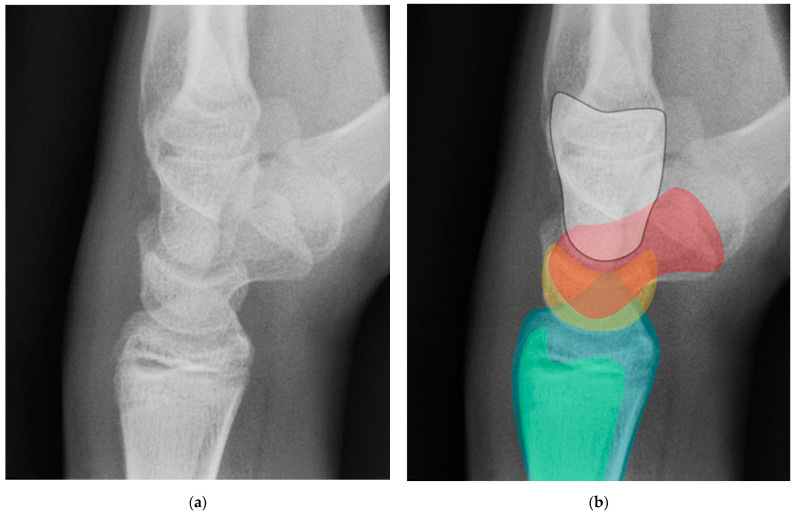
(**a**) Neutral lateral view wrist radiograph showing normal anatomy; (**b**) Radiograph overlay depicting its constituent anatomy; Green—ulna, Blue—radius, Yellow—lunate, Red—scaphoid, White—capitate.

Normal alignment of the carpus should always be assessed on neutral lateral view (Figure 18), as previously described [6]. In a normal wrist, a line can be drawn down the long axis of the radius that passes through the lunate, capitate, and metacarpals, with the radius cupping the lunate and the lunate cupping the capitate in a pillar-like fashion. Even with small amounts of projectional rotation, the biomechanical pillar along this line should remain intact. It is important to note that these intercarpal angles are only accurate on a “true” lateral view, when the pisiform is observed between the ventral surface of the capitate and distal pole of the scaphoid, ideally at the midpoint between these two structures. 

**Figure 19:**
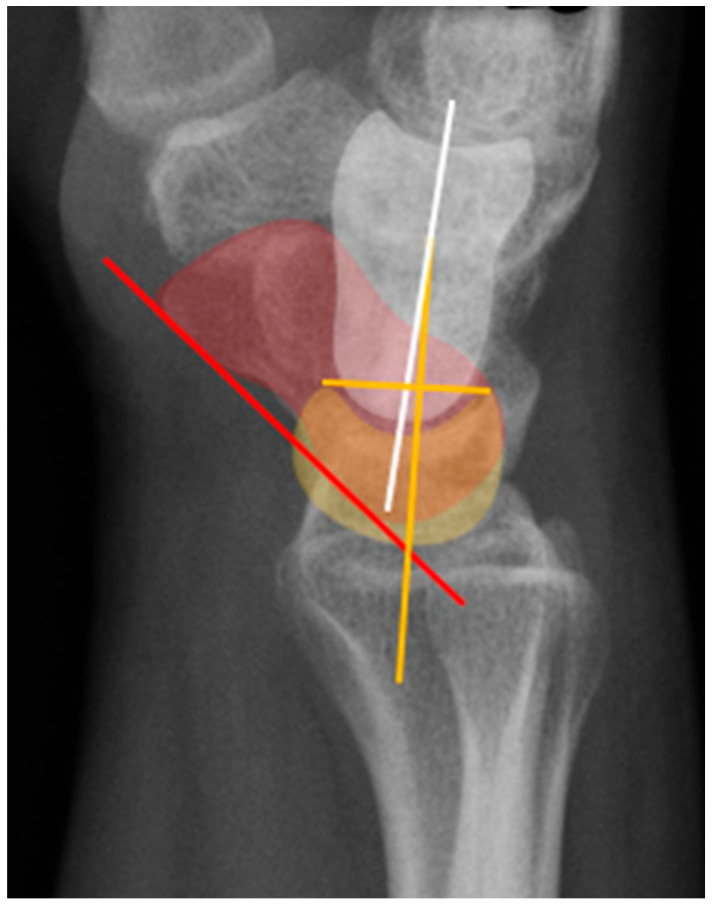
Illustration of the normal carpal alignment and angles. The scapholunate angle is formed between a line drawn along the volar surface of the scaphoid (red line) and a line through the axis of the lunate (yellow line). The capitolunate angle is formed between a line drawn through the axis of the capitate (white line) and a line through the lunate axis (yellow line). Capitate (white), scaphoid (red), lunate (yellow).

The scapholunate angle is formed between a line drawn along the axis or volar surface of the scaphoid and a line through the axis of the lunate, with the normal range being 30–60° (Figure 19) [3]. The capitolunate angle is formed between the lunate axis line and a line through the axis of the capitate, with a normal range of less than 30°, as these should be neutrally aligned (near 0°) in a normal wrist (Figure 19) [6,13].

The intrinsic ligaments of the wrist are deep, interosseous ligaments spanning between adjacent carpals. Many have components of both true ligaments (with collagenous fibers and fascicles) and functionally less important areas of fibrovascular or fibrocartilaginous tissue. These are intimately related to the extrinsic ligaments in most cases. The key intrinsic ligaments are the scapholunate ligament (SLL), the lunotriquetral ligament (LTL), and the scaphotrapeziotrapezoid ligament (STTL) [12]. 

**Figure 20:**
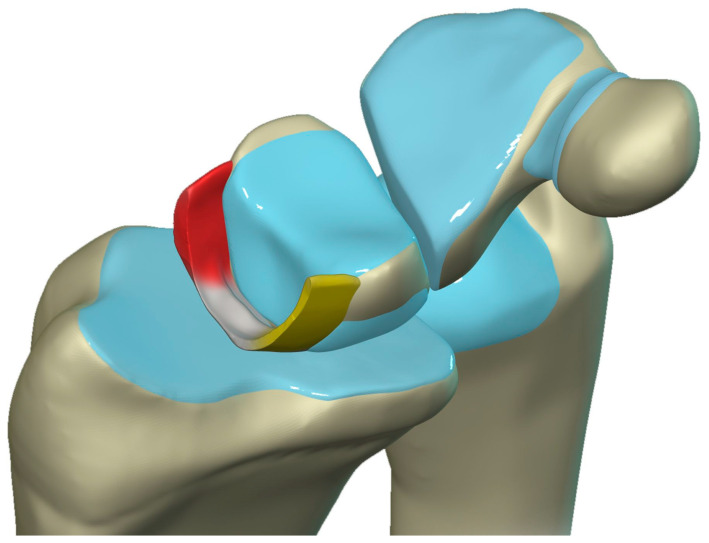
Illustration of the scapholunate ligament; Dorsal—red, Proximal—white, Volar—yellow.

**Figure 21:**
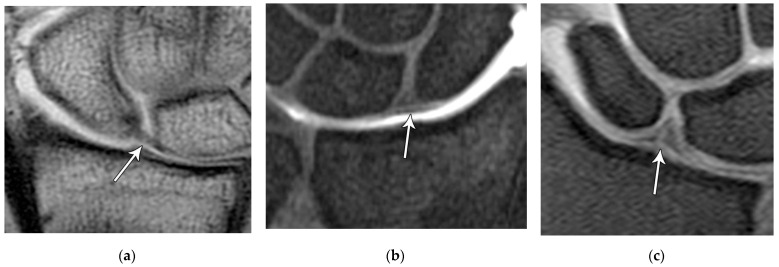
(**a**) Coronal T1 MR image showing a normal linear morphology of the proximal scapholunate ligament (SLL). (**b**) In a different patient, coronal PD FS MR image showing a normal triangular morphology of the proximal SLL. (**c**) In a different patient, coronal PD FS MR image showing an additional normal triangular morphology of the proximal SLL with a tear.

**Figure 22:**
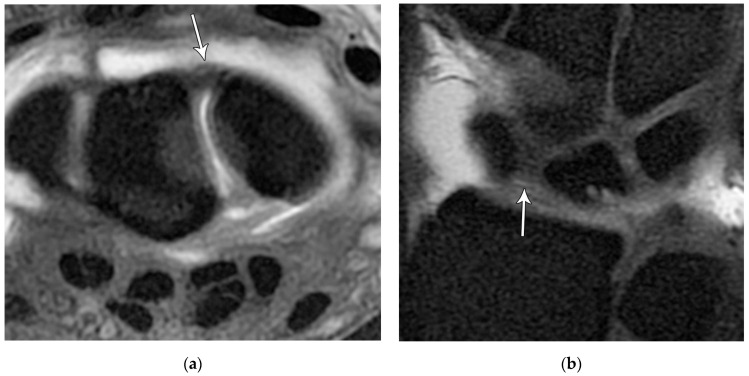
(**a**) Axial T1 FS MR arthrogram images showing a normal thick dorsal scapholunate ligament (arrow). (**b**) In a different patient, coronal T1 FS MR arthrogram images showing a normal thick dorsal scapholunate ligament (arrow).

The scapholunate ligament can be thought of as having three separate components: proximal, dorsal, and volar (Figure 20) [2,14]. Tears are often traumatic in etiology (e.g., fall on outstretched hand or FOOSH) and associated with symptoms. The proximal component is also known as the “central membranous component” due to its location and composition (Figure 21a). Partial tears or degenerative perforations of this membranous portion are common and usually asymptomatic. Typically, this portion has a triangular, meniscal projection distally (Figure 21b,c). The dorsal component is the thickest and has the most significant role functionally (Figure 22). The volar component is thin and weak and, therefore, is not of great biomechanical importance, and isolated injury is uncommon. 

**Figure 23:**
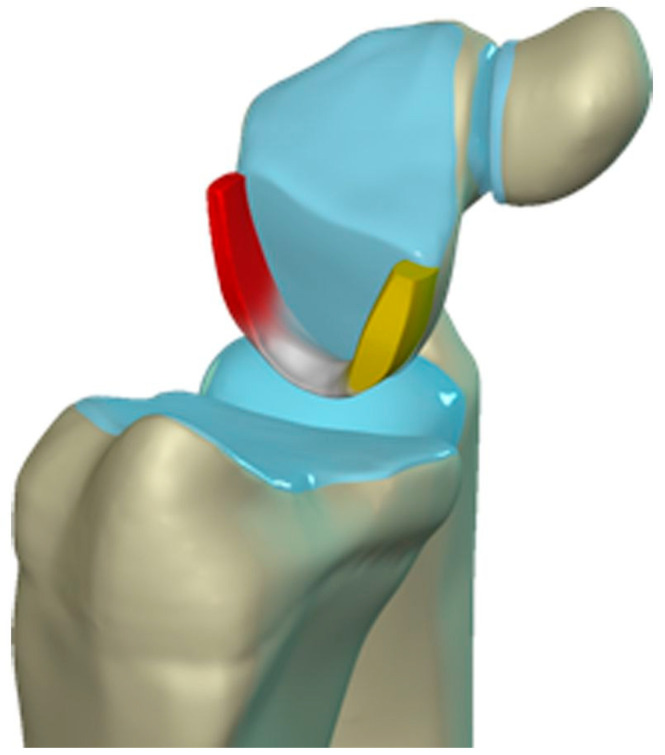
Illustration of the lunotriquetral ligament; Dorsal—red, Proximal—white, Volar—yellow.

**Figure 24:**
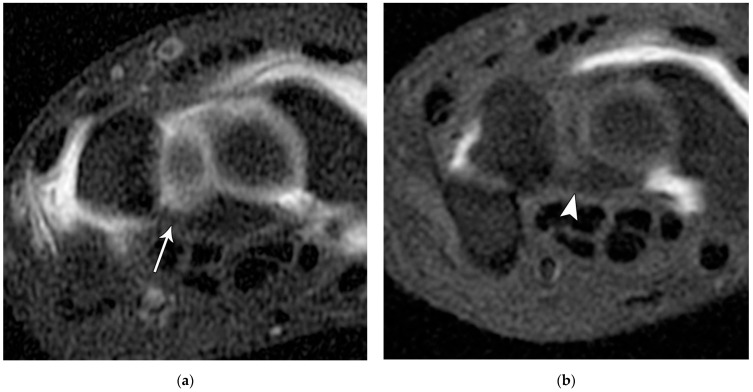
(**a**) Axial T1 FS MR arthrogram image showing a normal, thick volar lunotriquetral ligament (LTL) (arrow). (**b**) In a different patient, Axial T1 FS MR arthrogram image showing another example of the volar LTL fibers (arrowhead).

The lunotriquetral ligament can also be thought of as having three separate components as well: volar, dorsal, and proximal (Figure 23) [8,14]. The volar component is the thickest and most functionally important part of the LTL (as opposed to the SLL whose main structural fascicles are dorsal), limiting triquetral extension (Figure 24). The dorsal component of the LTL is thinner, but still more robust than the proximal membranous component which is again frequently triangular with a meniscal projection distally. The proximal membranous component has a range of MRI appearances that can be mistaken for tears or may not be detectable at all.

The extrinsic ligaments are those that arise from the radius and ulna and insert onto the carpal bones [25,26]. More attention is generally given to the intrinsic ligaments of the wrist by radiologists; however, the validity of that approach is questionable and full attention should be paid to the extrinsic ligaments as well. These ligaments are grouped as the dorsal capsular, volar radiocarpal, volar ulnocarpal, and volar midcarpal ligaments. 

**Figure 25:**
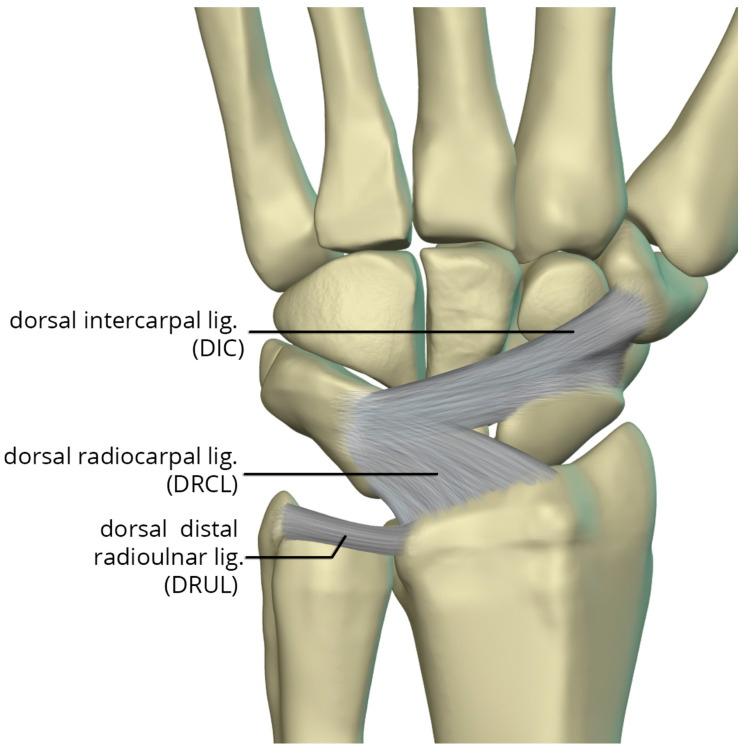
Illustration of the dorsal capsular ligaments: the dorsal radiocarpal ligament and dorsal intercarpal ligament.

The dorsal capsular ligaments (Figure 25) include the dorsal radiocarpal ligament (DRCL) and dorsal intercarpal ligament (DIC). The dorsal radiocarpal ligament (DRCL) is also referred to as the dorsal radiotriquetral ligament [26]. The DRCL extends from the dorsal rim of the radius, between Lister tubercle and the sigmoid notch, to the dorsal triquetrum. This ligament has fibers continuous with the fourth and fifth extensor compartment septa. Variants of this ligament include various additional bands, one of which may cover the dorsal aspect of the proximal scaphoid. The dorsal intercarpal ligament (DIC) is also sometimes referred to as the dorsal scaphotriquetral ligament due to its attachments. The DIC ligament extends from the dorsal ridge of the triquetrum to the dorsal groove of the scaphoid and proximal rim of the dorsal aspect of the trapezium. Along its course some fibers attach to the dorsal distal lunate. The DIC ligament may have one or two distinct fascicles, and sporadically has fibers extending to the trapezoid and capitate. 

**Figure 26:**
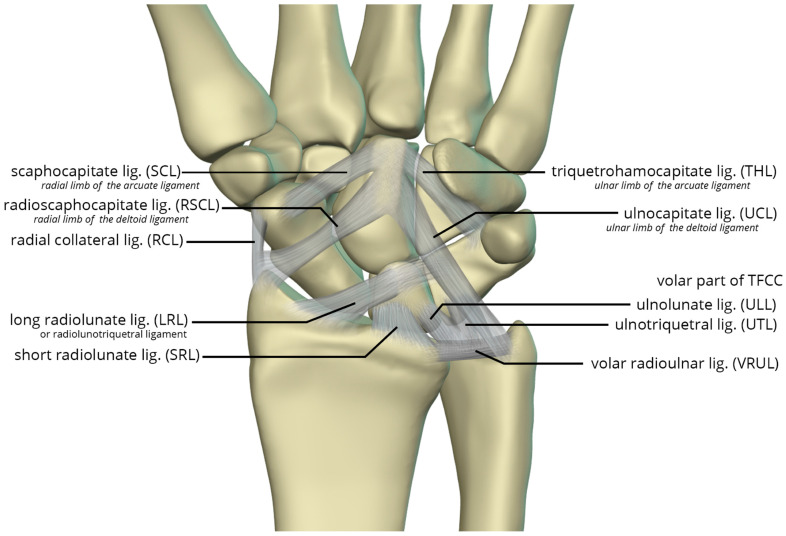
Illustration of the volar extrinsic carpal ligaments including the volar midcarpal ligaments, volar capsular ligaments, and volar ulnocarpal ligaments.

The volar radiocarpal ligaments include the radial collateral ligament (RCL), radioscaphocapitate ligament (RSCL), short radiolunate ligament (SRL), and long radiolunate ligament (LRL) (Figure 26). The RCL extends from the radial styloid to the scaphoid head. The RSCL arises from the radial half of the volar rim of the scaphoid fossa. The RSCL attaches to the capitate, forming a sling across the scaphoid. Some fibers may attach to the scaphoid. The SRL anchors the lunate from the radial half of the volar cortex of the lunate to the volar rim of the lunate fossa of the radius. The LRL arises from the volar aspect of the ulnar half of the scaphoid fossa rim of the radius. Distally, the LRL attaches to the volar aspect of the lunate, with some fibers also attaching to the volar triquetrum, and therefore may also be known as the radiolunotriquetral ligament. 

The volar ulnocarpal ligaments (Figure 26) include the ulnolunate ligament (ULL), the ulnotriquetral ligament (UTL), and the ulnocapitate ligament (UCL) [25,26]. The ULL and UTL originate from the volar radiolunate ligament and attach distally to the volar surfaces of the lunate and the triquetrum, respectively. The UCL extends from the ulnar head to the capitate. The ULL and UTL, deeper to the UCL, is a single sheet of tissue arbitrarily divided according to their distal attachments.

Lastly, the volar midcarpal ligaments include the arcuate and deltoid ligaments that are further subdivided into radial and ulnar limbs. The scaphocapitate ligament (SCL) arises from the ulnar aspect of the distal pole of the scaphoid, deep to the RSCL, and extends to the volar capitate body. It represents the radial limb of the arcuate ligament. The triquetrohamocapitate ligament (THL) extends from the triquetrum to the capitate, across the hamate. It represents the ulnar limb of the arcuate ligament. Some describe this as the distal band of the palmar scaphotriquetral ligament, which is another mid-carpal capsular ligament superficial to the SCL and THL [26,27]. The distal RSCL represents the radial limb of the deltoid ligament, and the UCL constitutes the ulnar limb of the deltoid ligament, supporting the head of the capitate [27].

In general terms, carpal instability (CI) may be categorized as dissociative (CID), non-dissociative (CIND), combined/complex (CIC), or adaptive (CIA) [19]. These groupings permit additional explanation of injury patterns such as DISI and VISI. CID is the disruption of a normal relationship between carpals in the same row and can be thought of as intrinsic ligament failure. The proximal row CID is more common than the very rare distal row CID. Proximal row CID includes scapholunate dissociation (SLD) which is associated with DISI, and lunotriquetral dissociation (LTD) which is associated with VISI, unstable scaphoid fracture, and Kienböck disease. CIND is the disruption of the normal relationship between the distal and proximal carpal row (midcarpal pattern), or between radius and proximal carpal row (radiocarpal pattern). CIND can be thought of as an extrinsic ligament failure. CIC is a combination of these two, containing features of both CID and CIND, with disruption of a normal relationship between carpals in the same row and between rows. This includes perilunate dislocation and fracture-dislocation spectrum, isolated carpal bone dislocation, and axial carpal dislocations. CIA is defined as an altered carpal function as a result of extra-carpal pathology, where altered carpal postures are not due to injury to the carpus itself. CIA includes Madelung deformity and distal radial fracture with malunion as an example [21].

The various patterns of carpal instability exist as a spectrum which ranges from frankly obvious instability (acute fracture-dislocation) all the way to nearly normal with only a clinical complaint and normal imaging and physical exam. The Watson classification categorizes carpal instability in relation to imaging in five stages. Stage 0 is termed ‘normal’, described as an asymptomatic patient with normal function of the carpus. Stage 1 is termed ‘pre-dynamic’, described as wrist pain with symptoms of instability; however, the imaging is normal. Stage 2 is termed ‘dynamic’, described as pain with dynamic or stress imaging demonstrating an imaging abnormality. Stage 3 is termed ‘static’, described as pain with abnormal imaging without stress. Stage 4 is termed ‘posttraumatic arthritis’, which includes scaphoid nonunion advanced collapse (SNAC) and scapholunate advanced collapse (SLAC) [28].

**Figure 27:**
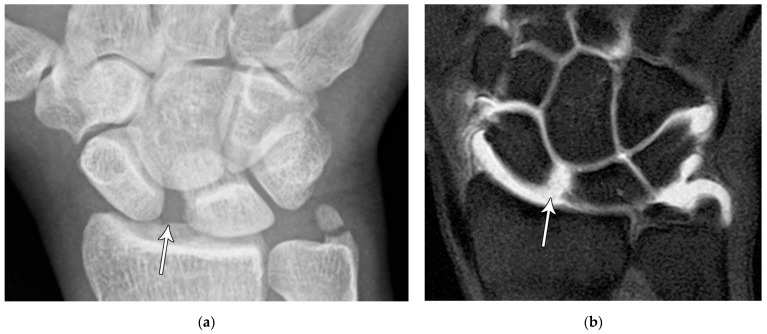
(**a**) PA wrist radiograph showing scapholunate joint widening (arrow). (**b**) Coronal T1 FS MR arthrogram in a different patient showing disruption of the scapholunate ligament (arrow).

**Figure 28:**
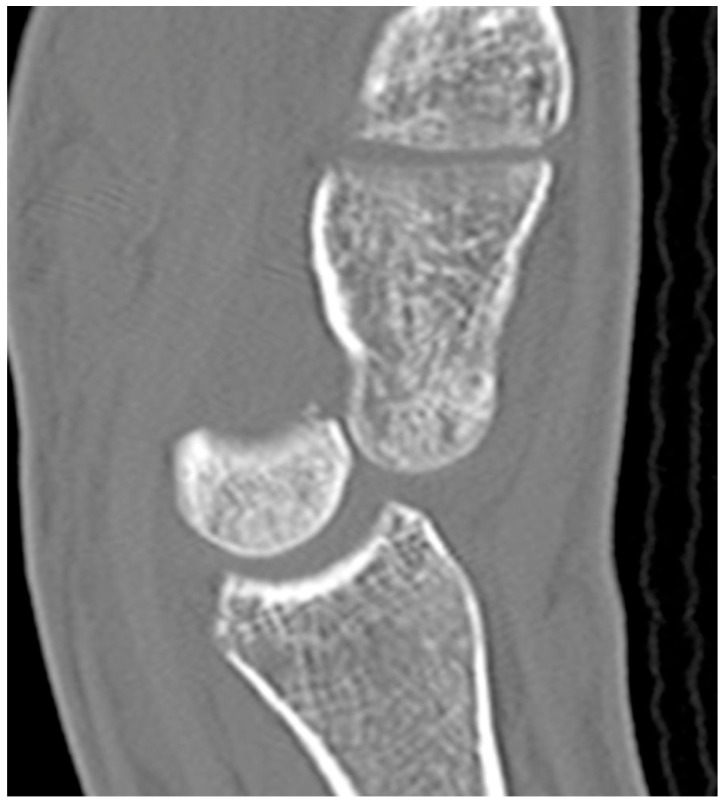
Sagittal CT wrist showing dorsal dislocation of the capitate in stage two perilunate dislocation.

**Figure 29:**
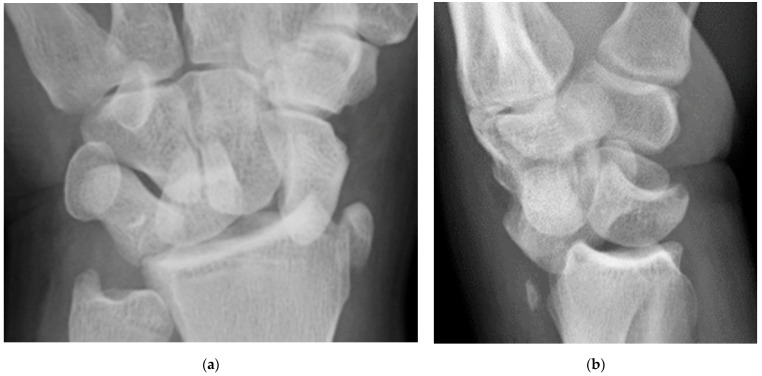
(**a**) PA wrist radiograph showing scapholunate dissociation, lunotriquetral dissociation, and radial styloid avulsion. (**b**) Lateral radiograph showing capitate dislocation. In addition to the PA radiograph, findings are consistent with a midcarpal dislocation, stage three perilunate injury.

**Figure 30:**
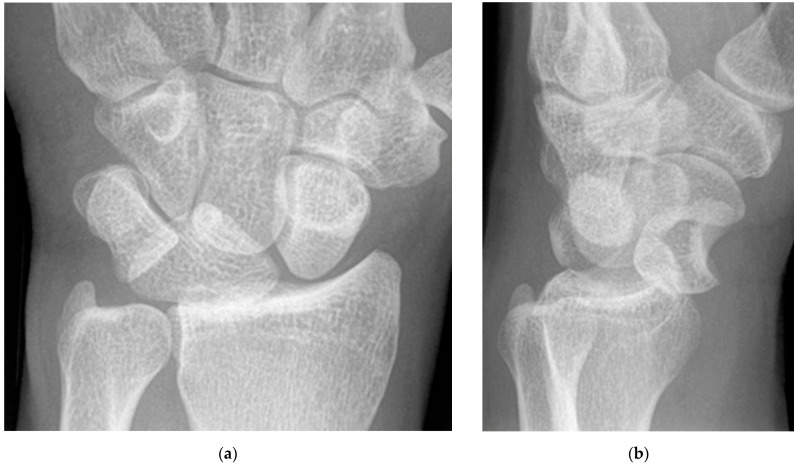
(**a**) PA wrist radiograph showing the “piece of pie sign” of a dislocated lunate. (**b**) Lateral wrist radiograph showing an anteriorly dislocated lunate which is anteriorly rotated ~90°.

Perilunate injury involves both intrinsic and extrinsic ligaments and results in a fairly predictable order of progression when the wrist is loaded in extension, ulnar deviation, and intercarpal supination. The typical history is a fall onto a dorsiflexed wrist (e.g., the FOOSH), and the injury progresses through four distinct stages depending on the magnitude of the traumatic force that it sustained. The first stage consists of scapholunate dissociation (Figure 27a). As the scapholunate joint is disrupted, the scapholunate and radioscaphocapitate ligaments are injured, causing volar scaphoid rotation with or without dorsal rotation of the lunate, and scapholunate joint widening (Figure 27b). The second stage consists of perilunate dislocation (Figure 28). In this stage, the scapholunate injury found in the first stage is still present, but as the force is increased, this trauma has extended to involve the capitolunate ligament, which then allows the capitate to be dislocated. The third stage is again a progression of the prior stages in which the lunotriquetral ligament is disrupted in addition to the findings of the second stage. Neither the capitate nor lunate is aligned with the distal radius, often referred to as a midcarpal dislocation. There is also interval development of widening/malalignment of the lunotriquetral and triquetrohamate joints, which is frequently associated with a volar triquetral fracture (Figure 29). The fourth and final stage of injury results in tears/disruptions of all the ligaments surrounding the lunate, including the dorsal radiocarpal ligament, resulting in palmar dislocation of the lunate (lunate dislocation). On PA radiographs, the lunate shows the classic “piece-of-pie” sign, and on the lateral view, there is a “tipped teacup” appearance (Figure 30). 

**Figure 31:**
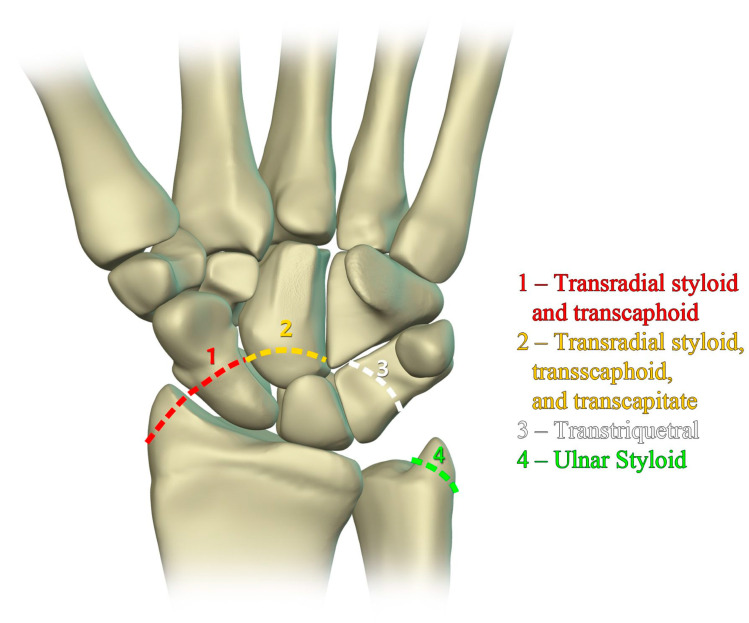
Illustration of greater arc injuries and their trans-osseous variants that can occur in conjunction with perilunate injury.

The previously mentioned ligamentous perilunate injuries are considered “lesser arc” injuries, but each stage may occur as a greater arc injury, which has a trans-osseous (or equivalent) path of biomechanical failure (Figure 31) instead of the previously described transligamentous path. Stage 1 injury is divided into transradial styloid and transscaphoid injuries. In transradial styloid injury, a large radial styloid fragment may be seen as the result of avulsion of the radioscaphocapitate ligament, which is itself typically injured in the lesser arc pattern. Stage 1 transscaphoid injury constitutes approximately 95% of greater arc injuries as this is simply a scaphoid fracture (usually the waist), and the proximal pole maintains its relationship to the lunate with an intact SLL. In stage two injury, a small radial styloid fragment from a radial styloid tip fracture may be seen as a result of avulsion of the radial collateral ligament. The distal pole of the scaphoid moves dorsally with the capitate and as the capitate dislocates, the dorsal lip of the radius or lunate may act as a chisel, causing a transverse fracture of the waist or proximal pole of the capitate. In stage three injury, triquetral body fracture and avulsion of the LRL and/or UTL may occur. The classic dorsal avulsion of the common attachment of the dorsal extrinsic ligaments seen in isolated triquetral fractures is not typically associated with this injury. An ulnar styloid fracture is commonly associated and may be seen with any stage of injury [11].

Management of stage two and three perilunate dislocation consists of immediate closed reduction in which axial traction is used and posteroanterior pressure applied while the hand moves from extension to flexion to bring the capitate back into the lunate fossa. An audible clunk usually signifies reduction. Management of stage four injuries consists of closed reduction with axial traction and anteroposterior pressure applied while the hand moves from flexion to extension to bring the lunate back into the lunate fossa between the capitate and radius [22]. Of note, if the lunate is rotated >90°, this maneuver may compromise the vascularity to the lunate, as the lunate vascular supply may solely come through the vascular bundle within the short radiolunate ligament. Operative management occurs after 3–5 days after closed reduction so that swelling can subside to the point where corrective surgical repair of ligamentous injury can be performed in addition to percutaneous fixation using Kirshner wires to maintain alignment [21].

**Figure 32:**
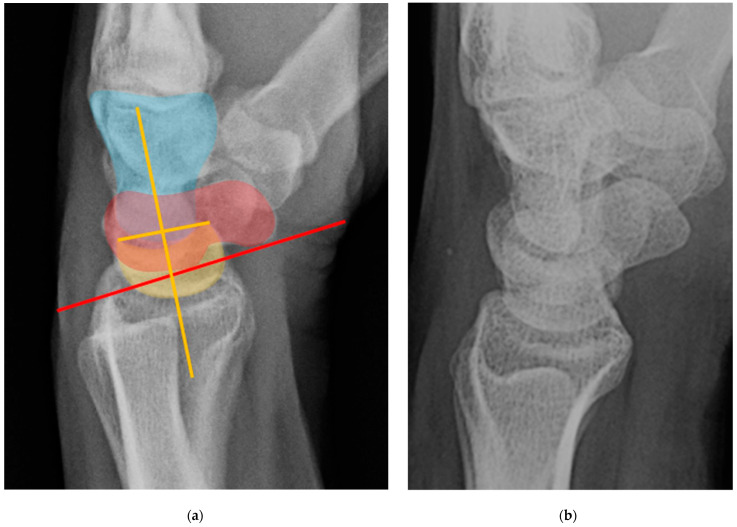
(**a**) Lateral wrist radiograph with overlay showing excessive volar rotation of the scaphoid relative to the lunate, with an increased scapholunate angle to nearly 90°. The red line is drawn along the volar surface of the scaphoid while a yellow line is drawn through the lunate axis; (**b**) Normal lateral wrist radiograph for comparison. Capitate (blue), scaphoid (red), lunate (yellow).

Dorsal intercalated segment instability (DISI) occurs when there is disruption of the SLL [23]. The “intercalated segment” can be considered a bone or series of bones fixed in a passive position between other active motion segments [4]. Because the proximal carpal row including the scaphoid, lunate, and triquetrum has no tendinous attachments, its motion is solely passive, dependent on the forces applied through its surrounding constraints. DISI deformity begins when the SLL loses its integrity. Because the posterior fibers of SLL are the thickest, it is the main dorsal stabilizer restricting dorsal rotation of the lunate relative to the capitate, and the volar scaphoid rotation. The rupture of the SLL disrupts the stabilizing balance and results in this malalignment pattern. On a lateral radiograph of a normal wrist, the scapholunate angle measures between 30° and 60° as the scaphoid and lunate are both held upright by the SLL. After SLL rupture, the scapholunate angle widens to greater than 60° due to excessive volar rotation of the scaphoid relative to the lunate (Figure 32) often with eventual dorsal rotation of the lunate as well [8,21]. Management of DISI and acute tears of the SLL consists of surgical repair with suture anchors and temporary K-wire fixation.

**Figure 33:**
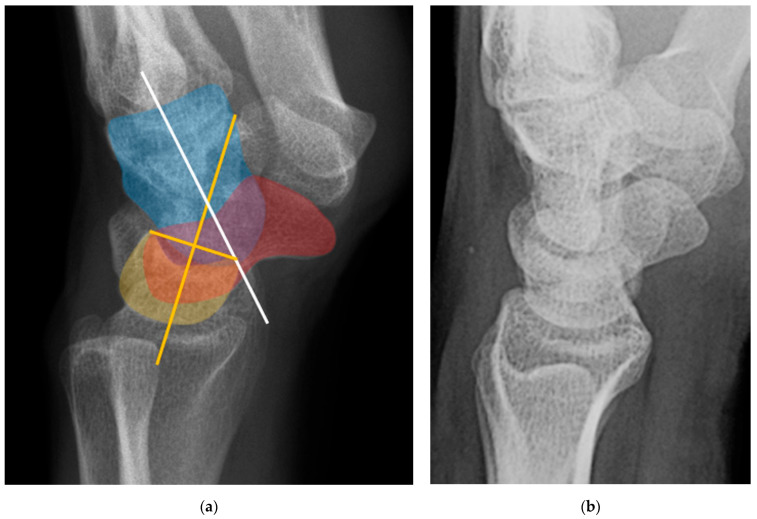
(**a**) Lateral wrist radiograph with overlay showing excessive volar rotation of the lunate relative to the capitate, with an increased capitolunate angle beyond 30°. The white line is drawn through the capitate axis while a yellow line is drawn through the lunate axis. Capitate (blue), scaphoid (red), lunate (yellow). (**b**) Normal lateral wrist radiograph for comparison.

Similar to the role that disruption of the SLL plays in DISI, volar intercalated segment instability (VISI) deformity occurs when there is disruption of the lunotriquetral ligament (LTL) [23]. Because the anterior fibers of LTL are thickest, it is the main volar stabilizing element restricting the lunate from volar rotation relative to the capitate [4]. Rupture of LTL causes the capitolunate angle to increase beyond the normal range of less than 30°, in the opposite direction as seen with DISI. Because the scaphoid is usually still tethered to the lunate in VISI, it rotates in a volar direction with the lunate. On a lateral radiograph of a normal wrist, the capitolunate angle measures less than 30°, and if the angle measured greater than 30°, it would represent LTL rupture and VISI (Figure 33). VISI is considered a CID lesion when there is a traumatic etiology and CIND lesion if rheumatic. When this occurs with perilunate injury, it is classified as a CIC lesion as it involves ligaments and/or bone from proximal and distal rows. Management of VISI consists of early reduction and casting, closed reduction and fixation with K-wires, or capitolunate fusion.

**Figure 34:**
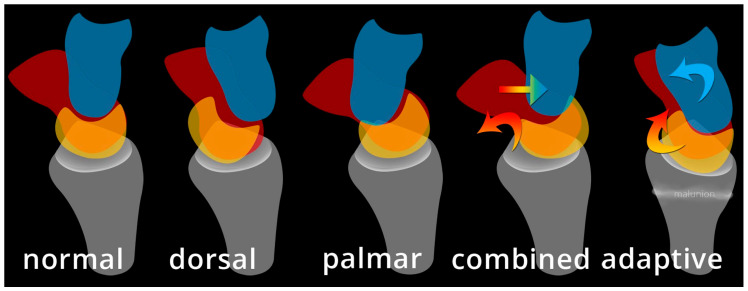
Illustration depicting the different variants of midcarpal instability (midcarpal CIND). Capitate (blue), scaphoid (red), lunate (yellow).

Midcarpal instability (MCI) is a confusing, uncommon and somewhat poorly understood topic with a wide spectrum of information regarding its etiology, mechanism, terminology, and treatment [29]. It is a form of CIND, with instability between proximal and distal carpal rows. One way of approaching MCI is dividing it into extrinsic and intrinsic categories (Figure 34). Extrinsic MCI is described as adaptive instability. When there is angular change at the radius (usually with fracture of the distal radius), the volar carpal ligaments become lax and eventually fail to properly shift the proximal carpal row when the wrist ulnar deviates [30]. The classic example of this is the dorsal tilt of the distal radius with the extension of the proximal carpal row and flexion of the distal row. Treatment is performed with a corrective osteotomy to re-affirm stability in the carpus.

Intrinsic MCI is further broken down into palmar, dorsal, and combined (Figure 34). In palmar MCI, the head of the capitate translate anteriorly, and the proximal carpal row is flexed [29]. With ulnar deviation, the triquetrohamate joint engages, resulting in the proximal row snapping into extension. Treatment options of palmar MCI include conservative management, arthroscopic thermal capsulorrhaphy, soft tissue reconstruction, and a limited radiocarpal/intercarpal fusion depending on the structures involved. Dorsal MCI entails the subluxation of the capitate dorsally. Treatment of dorsal MCI includes conservative management, palmar ligament reefing, and/or dorsal intercarpal capsulodesis for stability. Combined intrinsic MCI has elements of both palmar and dorsal MCI, in addition to volar sag of the proximal row and dorsal subluxation of the capitate.

## 6. Conclusions

The role of imaging in patients with carpal fracture and instability has never been greater, continuing to be integral in the diagnosis and direction of care. Long-term outcomes are heavily tied to the radiologist’s ability to adequately identify and characterize the extent of the carpal instability and injury, ensuring the correct treatment plan is initiated, particularly in those that require corrective surgical management. With the goal of decreasing missed diagnoses and poor clinical outcomes, the importance of the radiologist’s comprehension of the different carpal instability patterns remains crucial.

## Data Availability

Not applicable.
